# A Matter of Metals: Copper but Not Cadmium Affects the Microbial Alpha-Diversity of Soils and Sediments — a Meta-analysis

**DOI:** 10.1007/s00248-022-02115-4

**Published:** 2022-09-30

**Authors:** Marco Signorini, Gabriele Midolo, Stefano Cesco, Tanja Mimmo, Luigimaria Borruso

**Affiliations:** 1 Faculty of Science and Technology, Free University of Bolzano, Piazza Università 5, Bolzano, Italy; 2 Department of Botany and Zoology, Faculty of Science, Masaryk University, Brno, Czech Republic; 3 Competence Centre for Plant Health, Free University of Bolzano, Bolzano, Italy

**Keywords:** Bacterial communities, Alpha-diversity, Soil, Sediment, Rhizosphere, Heavy metals, Meta-analysis

## Abstract

Heavy metal (HM) accumulation in soil affects plants and soil fauna, yet the effect on microbial alpha-diversity remains unclear, mainly due to the absence of dedicated research synthesis (e.g. meta-analysis). Here, we report the first meta-analysis of the response of soil microbial alpha-diversity to the experimental addition of cadmium (Cd) and copper (Cu). We considered studies conducted between 2013 and 2022 using DNA metabarcoding of bacterial and fungal communities to overcome limitations of other cultivation- and electrophoresis-based techniques. Fungi were discarded due to the limited study number (i.e. 6 studies). Bacterial studies resulted in 66 independent experiments reported in 32 primary papers from four continents. We found a negative dose-dependent response for Cu but not for Cd for bacterial alpha-diversity in the environments, only for Cu additions exceeding 29.6 mg kg^−1^ (first loss of − 0.06% at 30 mg kg^−1^). The maximal loss of bacterial alpha-diversity registered was 13.89% at 3837 mg kg^−1^. Our results first highlight that bacterial communities behave differently to soil pollution depending on the metal. Secondly, our study suggests that even extreme doses of Cu do not cause a dramatic loss in alpha-diversity, highlighting how the behaviour of bacterial communities diverges from soil macro-organisms.

**Graphical abstract:**

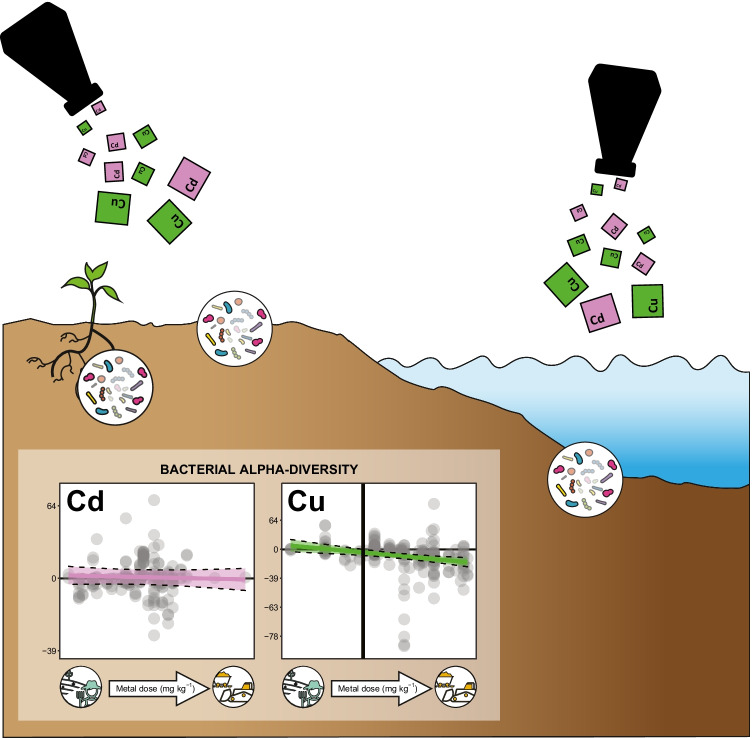

**Supplementary Information:**

The online version contains supplementary material available at 10.1007/s00248-022-02115-4.

## Introduction

The biodiversity measurement is based on three fundamental components: alpha- (local or within-sample), beta- (among-samples), and gamma- (biome-scale) diversity [1–5]. Alpha-diversity considers the number of species and/or their abundance distribution, and it is commonly used to characterize species community structure [6–9]. Alpha-diversity variability is usually associated with environmental perturbation and is widely used in biomonitoring to assess environmental health [10–13]. Specifically, ecosystems influenced by anthropic activities are generally characterized by lower levels of alpha-diversity due to habitat alteration and loss [14, 15]. Intensive agriculture and urbanization, pesticides, heavy metals (HMs) and other pollutants are among the major drivers of worldwide biodiversity decline [16–18]. Of the different biomes, soil and sediments are the final repository of these potentially toxic compounds, including HM, which are severely accumulated, posing a potential threat to fauna, plants and humans [19–21]. HM can be transferred to plants and animals throughout ecosystems in different ways, such as food web contamination (e.g. soil–plant-animal) or direct ingestion by the soil mesofauna [21–23]. When ingested by soil animals, HM can bioaccumulate in the body causing body size alteration and population size declines (e.g. earthworms) [24, 25]. Generally, the biological organization of soil invertebrates at the community level is also negatively impacted by HM [26, 27]. For example, community structure and species richness alterations strongly related to soil HM content have been observed in Oribatida and Mesostigmata mites [28]. The community structure of nematodes is also strongly altered by HM pollution, and the diversity of major nematode trophic groups decreases along a pollution gradient [29, 30].

While the role of HM in declining soil fauna species richness is generally clear [28, 31], their effects on soil bacterial alpha-diversity diverge [32, 33]. One gram of soil is typically inhabited by more than 4 × 10^3^ bacterial species [34–38], and this considerable diversity plays a pivotal role in ecosystem services involved in, to name a few, the biogeochemical cycling of elements, soil formation and interaction with macro-organisms (i.e. plants, soil fauna) [39–43]. Furthermore, freshwater and marine sediments are considered to be hotspots of bacterial diversity [44–46]; the top 50 cm of marine sediments can contain ~ 1 × 10^29^ bacterial species [47–50].

Previous investigations of polluted soils and sediments suggested that bacterial and fungal communities can exhibit differences in their response to HM [51–55]. This is partly based on general distinctions in biochemical pathways activated by bacteria and fungi in response to HM [56–59]. In addition, there are cases where experimental conditions could inherently contribute to the estimation of HM ecotoxicity, limiting the possibility of extending conclusions to a broader ecological scale [59]. Indeed, recent investigations have reported negative, neutral and positive estimated responses of the richness of bacterial taxa to HM exposure [60–63]. There are various, debated, hypotheses to explain these contrasting results [59]. Many studies are not comparable due to differences in soil and experimental conditions, background pollution levels and other experimental covariates that influence the response of microorganisms to HM [64]. Furthermore, some studies consider only a small number of replicates, limiting robust ecological conclusions [65–67], or only specific soil ecosystems (i.e. vineyards, orchards, arable lands or grasslands) [68].

After an accurate selection of studies and the rejection of fungal entries due to insufficient existing research from 2013 to 2022, we carried out a large-scale meta-analysis investigating bacterial alpha-diversity responses to HM (Cd and Cu) contamination in soils and sediments along a gradient ranging from 0.3 to 3837 mg kg^−1^. The study aims to clarify (a) the relationship between the dose of HM addition and the response of microbial alpha-diversity, (b) if microorganisms respond differently in different environments (i.e. soil, rhizosphere, and sediments) and (c) if the chemical properties of the specific environment (pH and Organic Matter (OM) content) influence the direction and magnitude of the alpha-diversity response to HM.

## Methods

### Data Selection and Collection

We conducted a meta-analysis collecting data from primary papers reporting experimental results on the response of bacterial diversity to lab-scaled and controlled Cu and Cd additions in soils and sediments. A preliminary search was conducted to understand the quantity of studies for each HM. The literature on HM is highly heterogeneous, focusing not only on different environments (e.g. soil, sediment, freshwater and marine environments) but also on different approaches, from descriptive biomonitoring experiments to pollution experiments and those focusing on remediation of historically polluted sites.

Based on our search, we found that the only metals eligible for contrast-based meta-analysis (i.e. experiments using replicated treatments and control samples) were Cd and Cu (e.g. most studies of arsenic focused on single-species toxicological experiments rather than an extensive and exhaustive bacterial community analysis). At first, we focused on Cu and Cd due to the environmental issues related to their intensive usage (e.g. mining, agriculture, industrial wastewaters) [69–72]. Moreover, from the preliminary search for other HM eligible for being included in the meta-analysis, we found that only Cu and Cd have been sufficiently studied in compliance with the selection criteria we adopted. First, we searched Scopus and Web of Science databases (last access in January 2022). The search strings contained Boolean statements, e.g. ‘OR’ and ‘AND’, combining key search terms such as ‘bacterial diversity’ and ‘copper’ or ‘cadmium’ and other additional terms (Supplementary Table S1). The search was restricted to peer-reviewed articles published between 2013 and 2021, as primary papers published after 2013 were more likely to adopt metabarcoding and next-generation sequencing (NGS) techniques for bacterial ecology analysis. This approach resulted in a selection process starting from an initial 10,687 unique entries for which the title and abstract were screened to remove non-relevant studies. Of this initial number, after this first screening, 374 relevant primary papers were identified.

We further evaluated the eligibility of these primary papers using the following criteria: (1) the study was based on the addition of Cu or Cd and replicated treated samples were compared to replicated control samples; (2) the bacterial communities were inferred through amplicon sequencing of the 16S rRNA gene (bacteria) and ITS region (fungi) as taxonomic barcodes since comprehensiveness and homogeneity of databases are also required for reliable comparison; (3) both gradual or abrupt HM addition was considered, but the primary papers had a metal-free control to calculate contrast-based effect size (i.e. the log-response ratio) [73]. After applying the selection criteria, 54 primary papers were eligible for the meta-analysis that reported direct relationships between HM addition and variation in soil bacterial diversity indices. From these, only those with proper alpha-diversity data were retained (some papers did not report sufficient information to calculate diversity indices).

**Fig. 1:**
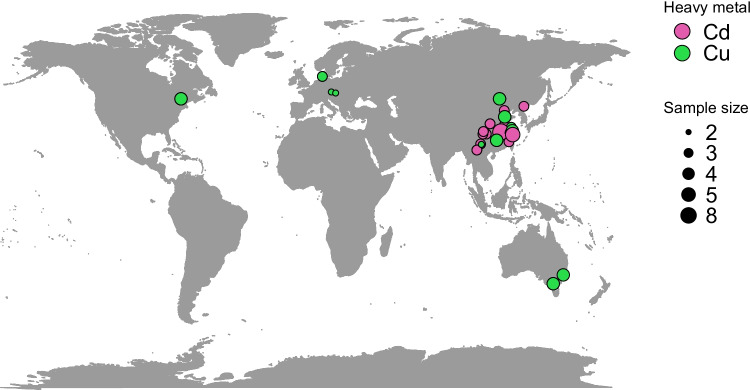
Locations of the experimental sites included in the meta-analysis. Points are coloured according to the metal used in the experiment and are weighted (point size) according to the sample size in the experimental design

The remaining 32 primary papers selected for our meta-analysis were mainly conducted in the People’s Republic of China (25) and a small number in Europe (2), North America (1) and Australia (2) (Fig. 1; PRISMA workflow shown in Fig. S1).

We extracted metal doses and alpha-diversity data directly from manuscripts, both from tables and figures. We further extracted additional information regarding the soil environment, the type of metal, the bacterial communities investigated, the environmental conditions possibly modifying the outcome of addition (soil pH and OM), the sample size and the time after treatment waited for the collection of samples.

Sample sizes in the selected papers were variable, ranging from 2 to 8 while most adopted an experimental design conducting metabarcoding analyses on 3–4 replicates per sample. HM additions spanned from 0.3 to 3837 mg kg^−1^. The maximal doses reached by Cd and Cu were respectively 1770 to 3837 mg kg^−1^. The response of bacterial communities to these addition ranges was measured over time, with sample collection occurring from 2 h to over 5 years after addition.

### Calculation of the Effect Size

The effect of HM addition on bacterial diversity metrics was calculated, for each case study, as the natural logarithm-transformed (ln) response ratio (Eq. 1) [73]:

1 $${\mathrm R\mathrm R}_{\mathrm i}=\mathrm{\mathrm l\mathrm n}\left(\overline{\mathrm{\mathrm X}}{\mathrm t }_{\mathrm i}\right)-\mathrm{\mathrm l\mathrm n}\left(\overline{\mathrm{\mathrm X} }\mathrm c\right)$$

where $\overline{X }$ t and $\overline{X }$ c are the average bacterial diversity values for treatment and control (metal-free) samples, respectively. Accordingly, we calculated multiple response ratios in cases where an experiment reported multiple treatments sharing the same control. Since this violates the assumption of independence among effect sizes, namely when multiple treated samples are compared to a single control group [74], the variance–covariance (VCV) matrix was computed to account for correlation of effect sizes, as proposed by [75]. In case the experiment was biomonitoring bacterial diversity over time, the metal-free control at the time zero was taken. The inverse of the sampling variance for each effect size in the VCV matrix was used to weight the meta-analysis. Sampling variance was calculated as follows, based on Hedges et al. [73] (Eq. 2):

2 $${\sigma }^{2} \left(\mathrm R\mathrm R\mathrm i\right)=\frac{{{\mathrm S\mathrm D}_{\mathrm t}}^{2}}{{\mathrm N}_{\mathrm t} {{\mathrm X}_{\mathrm t}}^{2}}+ \frac{{{\mathrm S\mathrm D}_{\mathrm c}}^{2}}{{\mathrm N}_{\mathrm c} {{\mathrm X}_{\mathrm c}}^{2}}$$

where SD is the standard deviation of the mean *X* (see Eq. 1), *N* is the sample size (i.e. the number of soil samples from which DNA was extracted, amplified and sequenced). When sample size was not reported, we imputed it from the median across all the primary papers included in the meta-analysis. The median of the sample size imputed this way was equal to 3 for both the treatment and control, for a total of 41 out of 502 observations that were missing the sample size. Similarly, in cases where the standard deviation of the mean was not reported, these were imputed by considering the coefficient of variation of the mean from all complete observations, following Hedges et al. [73].

### Data Analysis

Data were first split according to the microbial domain investigated, and then split according to the metal. Data were then analysed using a multilevel meta-analytic linear mixed-effect model using the *rma.mv* function within the *metafor* R package [76]. We first fitted a null model to estimate the weighted mean pooled effect size, namely the overall amount of alpha bacterial diversity changes across all experiments, independently from the dose of metal added.

Seven moderators were considered as influencing factors affecting bacterial community response in a meta-regression analysis: (a) diversity metrics considered in the primary papers (i.e. Shannon–Wiener, Simpson, observed OTU, Chao and ACE), (b) the ecosystem type where the experiment was conducted (sediment, soil or rhizosphere; categorical), (c) taxonomic kingdom (bacteria or fungi; categorical), (d) chemical form of the metal added (either in salt or nanoparticle form; categorical), (e) type of HM considered (either Cu or Cd; categorical), (f) soil pH and (g) soil OM content (% w/w of soil; continuous). Where no information on soil pH and OM content was retrievable from the primary papers (only 7 papers in which at least one of the two parameters was not available), geographic locations of the studies were used to access the SoilGrids database (at 250 m resolution) and extract estimates of pH (H_2_O) and OM data from 5 to 15 cm depth [77, 78].

Models were fitted with a crossed and nested random effect structure, as follows: (1|*study*/*experiment*/*id*), (*time* | *experiment*). The first component structure considers the possibility that the individual estimates within each observation (*id*) could be nested within the experiment and study grouping level [79]. The second component takes into account that the experiments could be nested within the time of sampling, considering an autoregressive correlation structure of true effects over time (this was achieved by setting struct = ‘CAR’ in the *rma.mv* function), since the results could have been collected at multiple time points within the same experiment [80, 81].

After computing the null model, we then fitted single meta‐regression models using the addition and the type of metal as the sole moderators, to compare changes among the metrics for a given amount of metal added to the matrices considered (i.e. rhizosphere, soil and sediment). Then, we fitted multiple meta‐regression models including other moderators and relevant interactions between the dose added and any other moderator since, in all the studies included, the dose was the sole discriminant factor between control and treated samples. We performed stepwise backward selection based on the Bayesian information criterion (BIC), selecting the model displaying the lowest BIC, excluding a moderator only if it was also dropped from the interaction term. Potential overparameterization of the model was checked by plotting the profile of the (restricted) log‐likelihood over the variance and the correlation components of the models [76].

Finally, publication bias was tested through visual examination of funnel plots and performing the Egger’s test for asymmetry by fitting null model residuals with the precision of the observations (1/SE) [82, 83]. Results of null model and publication bias are reported in Fig. S2 and Supplementary Table S2.

All analyses were performed in the R environment (version 4.2; R Core Team, 2022).

## Results

The meta-analysis was conducted on observations obtained by investigating bacterial communities since for fungi, the body of research that complied with our selection criteria was not sufficiently large to be included for quantitative analysis (see “Lack of Eligible Studies on Fungal Alpha-Diversity and HM”; Supplementary Table S2). Specifically, we ensured the robustness of obtained results by discarding all papers that did not focus on controlled additions of HM or did not rely on high-throughput sequencing to measure the response of soil microbial communities. This brought 32 final studies.

**Fig. 2:**
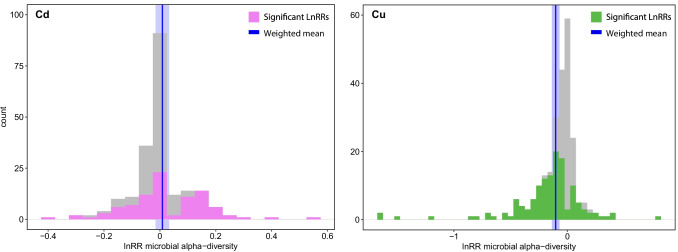
Distribution of the effect sizes of microbial alpha-diversity variation. Violet and green bars indicate the distribution of significant LnRRs (*p*‐value < 0.05) after respectively Cd and Cu addition. Blue vertical lines indicate the mean pooled estimate (solid line) with its 95% CI (shade) from a null mixed-effect model. Effect sizes reported consider alpha-diversity indices jointly

**Fig. 3:**
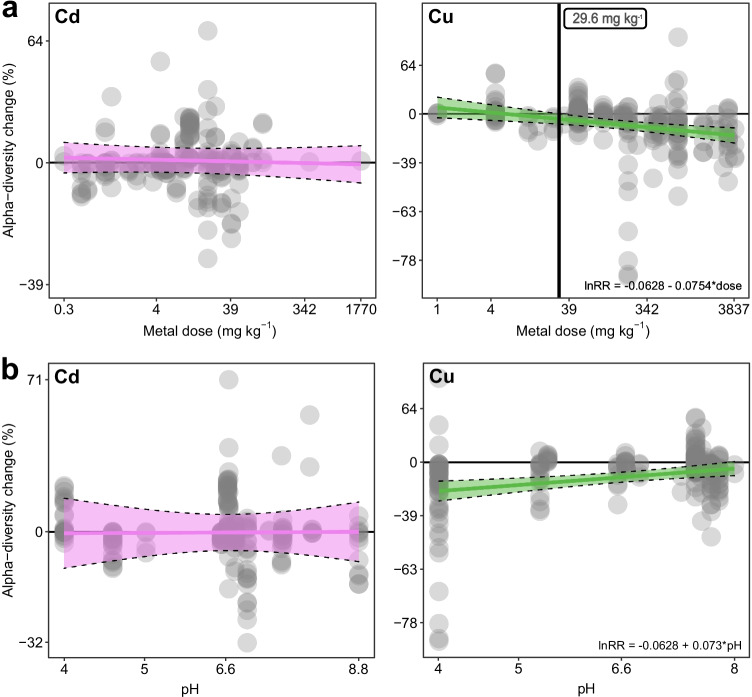
**a** Scatterplot showing dose–response relationship between alpha-diversity change (lnRR) and metal dose (on a log scale; back-transformed to mg kg^−1^ on the *x* axis, back-transformed to relative change (%) on the *y* axis). Cd on the left, Cu on the right. The horizontal black line indicates bacterial alpha-diversity LnRR effect size equal to zero. The meta-analysis revealed a dose–effect on bacterial alpha-diversity only for Cu. The vertical black line indicates the minimum alpha-diversity inhibitory dose corresponding to no overlap with zero by the 95% CI of the slope estimate (29.6 mg kg^−1^). **b** Scatterplot showing relationship between alpha-diversity change (lnRR) and the environment pH (on a log scale; back-transformed on the *x* axis, back-transformed to relative change (%) on the *y* axis). Cd on the left, Cu on the right. Effect sizes reported consider alpha-diversity indices jointly

We found that Cu addition caused a 9.81% mean pooled bacterial diversity loss (− 6.69 to − 12.82% with 95% CI) compared to untreated samples, conversely to Cd, that did not provoke any change in bacterial alpha-diversity (Fig. 2). The other experimental covariates did not influence the response of soil bacterial alpha-diversity to metal addition, except for the dose of Cu and pH (Final equation: LnRR ~ dose + pH) (Supplementary Table S4). For Cd, the model selection did not find any model explaining the effect size distribution better than the null model (Fig. 2, Supplementary Table S4). The multilinear mixed-effect meta-regression model finally obtained (i.e. the lowest BIC model obtained) accounted for a Cu-dependent dose–effect influencing the variation of bacterial alpha-diversity effect sizes across the studies (Fig. 3). The final model estimated a minimum inhibitory concentration of 29.6 mg kg^−1^ to register a significant bacterial alpha-diversity decrease (Fig. 3). From that minimum concentration upwards, the model fit indicated a linear decrease in alpha-diversity. For instance, at 100 mg kg^−1^, bacterial communities lost 5%, at 1000 mg kg^−1^ lost 11.1% and at 3837 mg kg^−1^, the bacterial communities investigated lost 13.89% of alpha-diversity (Fig. 3). The final model obtained for Cu contained pH as moderator of the effect size distribution as influencing increases in effect sizes moving from acidic to alkaline environmental conditions (lnRR =  − 0.0628 + 0.073 × pH).

Among the alpha-diversity metrics, we found no difference in their relative dose–response, except for richness that differed from the others (Simpson, Shannon–Wiener, Chao1 and ACE) (*p* = 0.0084) (Fig. 3). However, the same result was not recorded at the end of the BIC selection process; thus, this did not influence the dose–response.

## Discussion

### Bacterial Communities’ Metal-Dependent Response

Heavy metals, including Cu and Cd, are antimicrobial agents, and their activity has been tested on different bacterial species [84, 85]. Nevertheless, addressing the differential toxicity effect of Cu and Cd on bacteria is not straightforward. This is mainly due to the different direct and side effects that the two metals can cause to bacterial species [86]. Direct evidence pinpoints Cu as more detrimental for bacteria than Cd [84, 87]. For instance, iron-sulphur clusters of dehydratases (e.g., fumarase) are known as the main target of Cu in bacteria (Macomber and Imlay, 2009). On the other hand, for Cd, evidence showed that the toxicity of iron-sulphur clusters is less pronounced than that of Cu [88, 89]. Nevertheless, bacteria live in highly diverse and complex communities [90] in soils and sediments [91, 92]; thus, it is crucial to address HM toxicity at the community level rather than the effect on the single species. Our meta-analysis found that the response of soils and sediments bacterial alpha-diversity to HM is metal-dependent. Cu, differently from Cd, was correlated with a decrease in bacterial alpha-diversity (Fig. 2). While Cd-related response exploits mainly detoxification mechanisms (e.g., efflux, chelation etc.), Cu also causes significant oxidative stress to bacterial cells, forcing them to manage reactive oxygen species (ROS) and uncontrolled redox reactions in addition to metal detoxification [93, 94]. However, the bacteria alpha-diversity response to Cu was moderate (Fig. 3). Indeed, the dose must exceed 29.6 mg kg^−1^ to register an initial significant decrease in bacterial alpha-diversity (Fig. 3). The maximum loss of alpha-diversity (− 13.89%) was registered at super-excessive HM additions peaking to 3837 mg kg^−1^ (Fig. 3). The dose we registered are far beyond the levels of HM loads registered worldwide [95–98]. European topsoils display on average 60–100 mg kg^−1^ of Cu and 0.5–1 mg kg^−1^ Cd, similar to soils in other continents [72, 95, 99–101]. Exceptional concentrations coherent with our results can be found in specific intensive agricultural areas and mining sites where these values reach approximately thousands of mg kg^−1^ [102–106].

The negative Cu-related response was generally moderate compared to the extreme doses added to soils and sediments, which contrasts with the response of macro-organism biodiversity to HM. Soil macro-organisms (e.g. plants, nematodes and wild bees) are severely affected by HM at doses even lower than those reported in our work. For instance, nematode alpha-diversity decreased from 0.7 to 10 mg kg^−1^ of Cd and 42.7 to 1000 mg kg^−1^ of Cu [30], with a loss of more than 13.89% of total biodiversity (the maximum decrease we registered). Likewise, the diversity of wild bee species is known to be severely reduced in locations with soils polluted with HM (20–350 mg Cd kg^−1^ soil; [107]). Nevertheless, microorganisms live in complex communities and have already been demonstrated more resistant than single species subjected to HM in toxicological studies (i.e. higher minimum inhibitory concentrations) [108]. For microorganisms, the abrupt exposure to HM may not necessarily severely compromise alpha-diversity levels, though beta-diversity may rapidly change [109–111]. In fact, the use of alpha-diversity measures has been debated [112], highlighting that alpha-diversity is meaningless without additional bacterial analysis investigating both metabolic and ecological responses (i.e. biomass, respiration, enzymatic activities, beta-diversity analysis, bacterial functional ecology and inter-kingdom relationships through ecological network analysis).

**Fig. 4:**
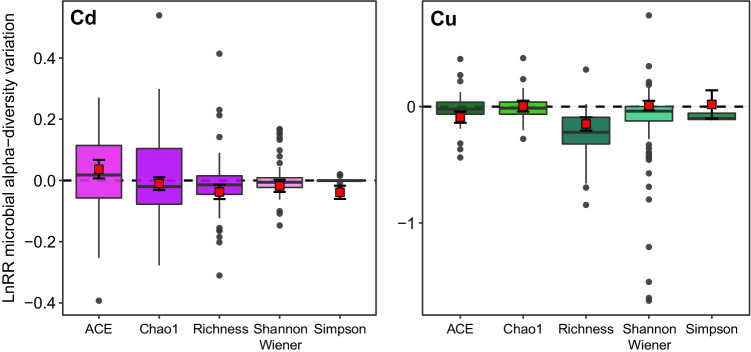
Distribution of the effect sizes according to the alpha-diversity metric considered. Cd-related effect sizes on the left, Cu-related ones on the right. The horizontal dashed line indicates bacterial alpha-diversity LnRR effect size equal to zero. No significant difference was detected across different diversity indices. Red square points indicate mean pooled effect (and 95% CI) obtained from mixed‐effect models using diversity metric type as predictor

Although alpha-diversity represents a specific component of biodiversity, it can be measured using different indices. In our work, both qualitative measures based on presence/absence (e.g. Chao 1, ACE, observed OTUs number) [113, 114] and quantitative measures based on taxon relative abundance (e.g. Shannon and Simpson) [115, 116] were recorded across the studies we considered. The synergic variation of diversity indices (i.e. Shannon–Wiener and Simpson) (Fig. 4) is explained by their mathematical relatedness in estimating diversity in bacterial communities. Contrarily, the discordancy between the Chao estimator and bacterial richness has different potential explanations. Indeed, there is evidence that the Chao estimator could fail in precisely estimating the correct level of richness of bacterial communities, being rather an accurate estimate of the lower bound of bacterial richness [117, 118]. Further, the decrease in richness may indicate a specialization of bacterial communities towards living in a metal-rich environment through metal-resistant species selection.

### Environment Conditions Might Influence the Bacterial Alpha-Diversity Response to HM

The toxicity of HM to soil organisms is partially dependent on the chemical composition of the surrounding matrix (e.g. pH, OM), which modulates HM bioavailability [19, 119]. By considering studies of different soil environments contaminated with HM in our analysis, we aimed to collect the largest possible soils and sediment matrix physical–chemical variability (i.e. pH 4–9, OM 1–8%). pH is one of the most important drivers of bacterial diversity [10, 120] and a crucial moderator of element availability in soil [121, 122]. Most complexed Cu is adsorbed to negative binding sites of dissolved organic matter. In acidic conditions, the protonation of these sites might cause a mobilization of adsorbed Cu and release to the soil solution [123, 124] where it can exert strong pressure over bacterial biomass [125, 126]. Indeed, pH has been already described as more important than total Cu content for its effect on bacterial communities [127]. Our study demonstrates that the response of bacterial alpha-diversity to Cu toxicity is not only dependent on the dose of Cu applied, but also on the pH of the soil/sediment (Fig. 2). The decrease in alpha-diversity of the bacterial communities subjected to Cu contamination is enhanced moving from alkaline to sub-acid, acid environmental conditions.

The recorded response of bacterial communities to HM did not vary across the soil environments investigated (i.e. bulk soil, rhizosphere, marine and freshwater sediments) (Supplementary Table S4). For instance, the response of bacterial community alpha-diversity to HM addition was not altered by the close proximity of plants. Although plants can modulate the local availability of HM in the rhizosphere, for example due to root exudation [128–130], their effect was likely masked by the extreme loads of Cu and Cd oversaturating the soil. Indeed, the modulation of HM availability by soluble organic ligands has been shown at low concentrations [131, 132].

### Lack of Eligible Studies on Fungal Alpha-Diversity and HM

Although, the crucial ecological significance of fungal communities (e.g. interaction with plants, construction of soil food-web, and carbon sequestration) [133–136], the number of works and consequently the available datasets regarding the effect of HM on their diversity is considerably lower than those for bacteria. Thus, we could not conduct a large-scale meta-analysis on fungi due to the limited number (*n* 6) of eligible works (Supplementary Table S2). Some authors have already postulated that bacterial and fungal communities could display differences in their response to HM [55]. There is evidence that fungal communities should be more resistant to HM [137], probably due to the different genetics and physiology characterizing the two kingdoms [55]. In addition, some authors hypothesized that, at least in short-term experiments of HM addition, fungi could benefit from carbon released by bacterial death cells, thus balancing the adverse effect of HM [55].

## Conclusions

We show that the response of bacterial alpha-diversity to Cd is absent. On the other hand, bacterial alpha-diversity decreases up to 14%, although extreme doses of Cu have been applied to soils and sediments (up to 3837 mg kg^−1^). We also demonstrate that bacterial communities respond differently to Cu addition in relation to soil and sediment pH, showing decreases in alpha-diversity in acidic conditions. In contrast to bacteria, we remark that more research should be conducted to explore the response of fungal communities to HM pollution in soils and sediments.

Our findings shed new light on the role of HM on microbial communities, unravelling a general overview of the response of microbial alpha-diversity to HM.

## Supplementary Information

Below is the link to the electronic supplementary material.

Supplementary file1 (DOCX 160 KB)

Supplementary file2 (R 19 KB)

Supplementary file3 (R 17 KB)

Supplementary file4 (CSV 116 KB)

## Data Availability

The dataset and R code are freely available at Figshare data repository (https://figshare.com) following this link: https://doi.org/10.6084/m9.figshare.19188917.v2.
