# Supplementary material for: A Matter of Metals: Copper but Not Cadmium Affects the Microbial Alpha-Diversity of Soils and Sediments — a Meta-analysis
Source: Microb Ecol. 2022 Sep 30;86(2):1071–81. doi: 10.1007/s00248-022-02115-4 (PMC10335967; doi:10.1007/s00248-022-02115-4)
Supplement: Supplementary file 1 — Supplementary file1 (DOCX 160 KB) [file 248_2022_2115_MOESM1_ESM.docx]

**A matter of metals: Copper but not cadmium affects the microbial alpha-diversity of soils and sediments, a meta-analysis**.

Marco Signorini* (1), Gabriele Midolo (2), Stefano Cesco (1), Tanja Mimmo (1-3) and Luigimaria Borruso* (1)

(1) Faculty of Science and Technology, Free University of Bolzano, Piazza Università 5, Italy;

(2) Department of Botany and Zoology, Faculty of Science, Masaryk University, Brno, Czech Republic;
(3) Competence Centre for Plant Health, Free University of Bolzano

*** Correspondence:**

Marco Signorini

[marco.signorini@natec.unibz.it](mailto:marco.signorini@natec.unibz.it)

Luigimaria Borruso

[luigimaria.borruso@unibz.it](mailto:luigimaria.borruso@unibz.it)

**Supporting information**

**Table S1:** Strings used for data collection

| **Organism** | **­­Metal** | **String** |
| --- | --- | --- |
| General | Copper | copper AND addition AND soil AND microbial AND diversity |
|  | Cadmium | cadmium AND addition AND soil AND microbial AND diversity |
| Bacteria | Copper | copper AND addition AND soil AND bacteria* AND diversity |
|  |  | copper AND (addition OR treatment OR amendment) AND soil AND bacteria* AND diversity |
|  | Cadmium | cadmium AND addition AND soil AND bacteria* AND diversity |
|  |  | cadmium AND (addition OR treatment OR amendment) AND soil AND bacteria* AND diversity |
| Fungi | Copper | copper AND addition AND soil AND fung* AND diversity |
|  |  | copper AND (addition OR treatment OR amendment) AND soil AND fung* AND diversity |
|  | Cadmium | cadmium AND addition AND soil AND fung* AND diversity |
|  |  | cadmium AND (addition OR treatment OR amendment) AND soil AND fung* AND diversity |

**Table S2:** Distribution of studies according to the domain investigated, the metal, and the dose added.

| study | observations | | metal | range of addition (mg/kg) |
| --- | --- | --- | --- | --- |
|  | **bacteria** | **fungi** |  |  |
| An et al (2019) | 12 | - | Cd | 0.6-1.2 |
|  | - | - | Cu | - |
| Asadishad et al (2018) | - | - | Cd | - |
|  | 8 | - | Cu | 1-100 |
| Cao et al (2020) | - | - | Cd | - |
|  | 8 | 8 | Cu | 150-450 |
| Cui et al (2019) | 2 | - | Cd | 393-1769 |
|  | - | - | Cu | - |
| Ding et al (2017) | 34 | - | Cd | 5-50 |
|  | - | - | Cu | - |
| Duan et al (2020) | 24 | - | Cd | 1-100 |
|  | - | - | Cu | - |
| Guan et al (2020) | - | - | Cd | - |
|  | 72 | - | Cu | 5-50 |
| Hou et al (2017) | 2- | - | Cd | 6 |
|  | - | - | Cu | - |
| Hu et al (2018) | 2- | - | Cd | 0.5-8 |
|  | - | - | Cu | - |
| Keblinger et al (2017) | - | - | Cd | - |
|  | - | 12 | Cu | 50-5000 |
| Kou et al (2018) | - | - | Cd | - |
|  | 2 | - | Cu | 34 |
| Li et al (2015) | - | - | Cd | - |
|  | 56 | - | Cu | 12.5-3200 |
| Li et al (2016) | - | - | Cd | - |
|  | 16 | - | Cu | 200-3200 |
| Li et al (2017) | - | - | Cd | - |
|  | 24 | - | Cu | 200-3200 |
| Lin et al (2021) | 2 | - | Cd | 5-25 |
|  | - | - | Cu | - |
| Liu et al (2021) | - | - | Cd | - |
|  | 9 | 9 | Cu | 50-200 |
| McTee et al (2019) | - | - | Cd | - |
|  | 8 | - | Cu | 174-2799 |
| Naveed et al (2014) | - | - | Cd | - |
|  | 16 | - | Cu | 175-3837 |
| Niu et al (2020) | 24 | - | Cd | 12 |
|  | - | - | Cu | - |
| Pu et al (2019) | - | - | Cd | - |
|  | 8 | - | Cu | 500 |
| Qiu et al (2020) | 4 | - | Cd | 2.5 |
|  | - | - | Cu | - |
| Sarria Carabalí et al (2020) | 24 | - | Cd | 20-40 |
|  | - | - | Cu | - |
| Sutcliffe et al (2019) | - | - | Cd | - |
|  | 8 | - | Cu | 46-487 |
| Wang et al (2020) | - | - | Cd | - |
|  | 2 | 2 | Cu | 16.18 |
| Wu et al (2018) | 12 | 12 | Cd | 5-20 |
|  | - | - | Cu | - |
| Yin et al (2019) | 24 | - | Cd | 0.5-20 |
|  | - | - | Cu | - |
| Zhang et al (2020) | - | - | Cd | - |
|  | 8 | - | Cu | 50 |
| Zheng et al (2018) | 6 | 6 | Cd | 0.3-30 |
|  | - | - | Cu | - |
| **Total observations** | **453** | **49** |  |  |

**Table S3:** Results of Egger’s regression test for publication bias. The test statistically verifies the asymmetry of funnel plots between effect sizes over their precision (inverse of standard error) through fitting a linear regression model. In our case, since the intercept does not differ significantly from zero, we conclude there is no asymmetry in the funnel plot and therefore there is no publication bias.

|  | **Estimate** | **Std. Error** | ***t*-value** | **Pr(>\|t\|)** |
| --- | --- | --- | --- | --- |
| (Intercept) | -5.473e-03 | 9.965e-03 | -0.549 | **0.583** |
| wi | 2.250e-05 | 2.173e-05 | 1.035 | 0.301 |
| Residual standard error: | | 0.218 on 500 degrees of freedom | | |
| Multiple *R*-squared: | | 0.00214 | |  |
| Adjusted *R*-squared: | | 0.0001438 | |  |
| *F*-statistic: | | 1.072 on 1 and 500 DF | | *p*-value: 0.301 |

**Table S4:** Best model selection. Model equations.

| Metal | Model name | Model formula | BIC | AIC |
| --- | --- | --- | --- | --- |
| Cd | final model | yi ~ NA | -382.78 | -402.81 |
|  | model 5 | yi ~ soil OM | -378.86 | -402.22 |
|  | model 4 | yi ~ dose + soil OM | -373.8 | -400.5 |
|  | model 3 | yi ~ dose + amended + soil OM | -368.46 | -398.5 |
|  | model 2 | yi ~ soil environment + dose + amended + soil OM | -358.85 | -395.57 |
|  | model 1 | yi ~ soil environment + dose + amended + soil OM + diversity metric | -341.77 | -391.83 |
|  | full model | yi ~ soil environment + dose + amended + soil pH + soil OM + diversity metric | -336.43 | -389.83 |
| Cu | final model | yi ~ dose + soil pH | 20.69 | -7.32 |
|  | model 3 | yi ~ dose + soil pH + diversity metric | 34.35 | -7.67 |
|  | model 2 | yi ~ dose + soil pH + soil OM + diversity metric | 39.62 | -5.89 |
|  | model 1 | yi ~ soil environment + dose + soil pH + soil OM + diversity metric | 48.08 | -4.44 |
|  | full model | yi ~ soil environment + dose + amended + soil pH + soil OM + diversity metric | 53.57 | -2.45 |

**
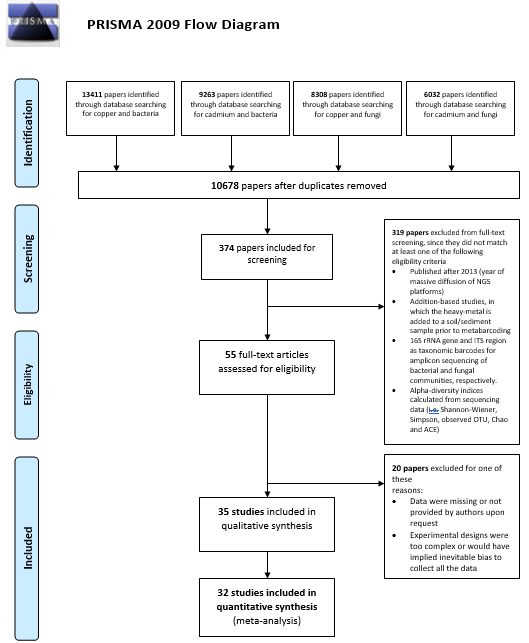
Fig. S1:** PRISMA 2009 Flow Diagram illustrating the paper selection process from initial literature screening to final papers included.

**Fig. S2:** Contour-enhanced funnel plot indicating the distribution of effect sizes over their precision (1/SE). Different levels of significance of the effect sizes are indicated by the shaded regions. The plot indicates no probable publication bias of the analysed data, since the effect size distribution is homogeneous within the statistically significant area (green). The plot of all results is shown on the left, while on the right is presented a subset of the results with the Lowest Inverse Standard Error.


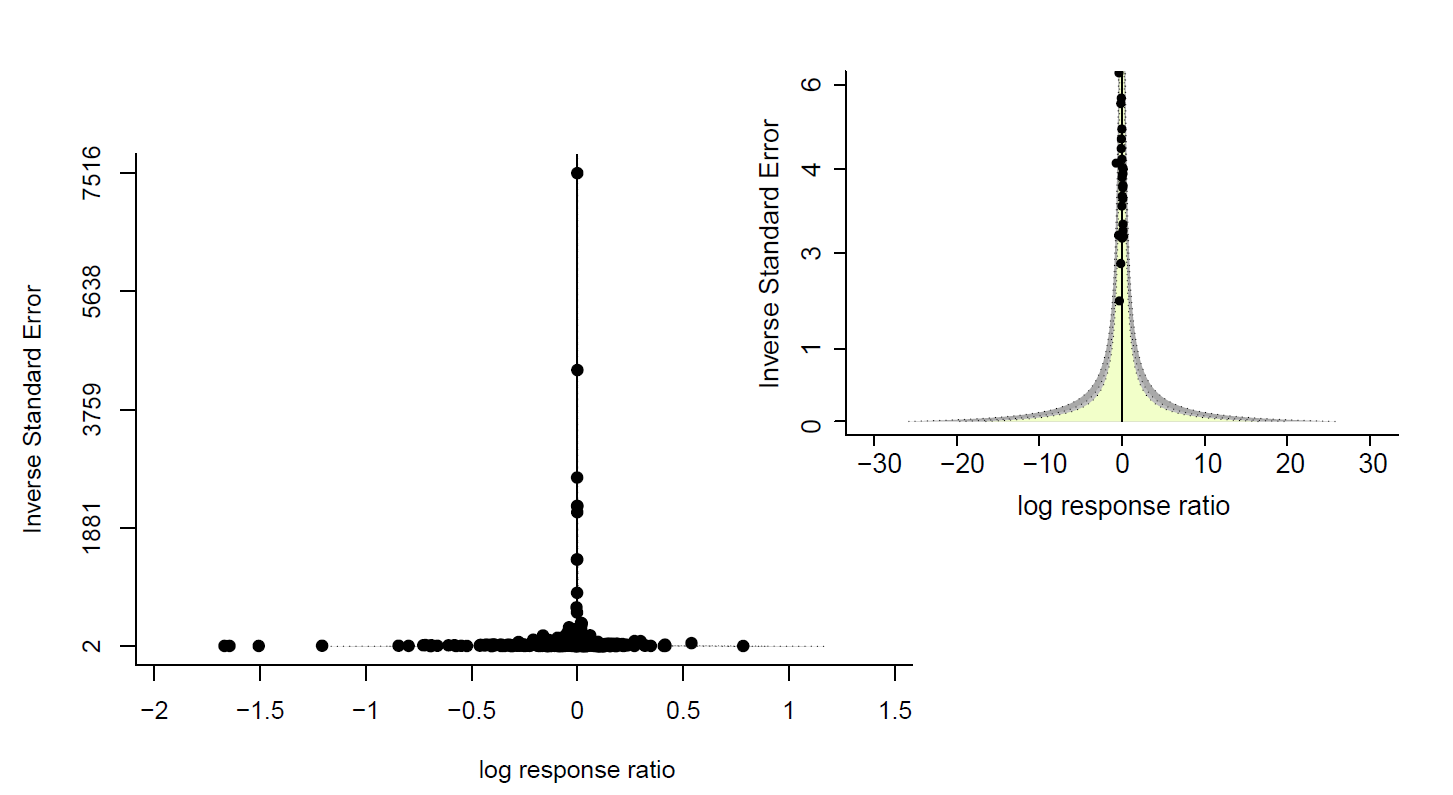


**Data sources**

An F., Li H., Diao Z. The soil bacterial community in cropland is vulnerable to Cd contamination in winter rather than in summer. (2019) *Environ Sci Pollut Res* **26,**114–125 https://doi.org/10.1007/s11356-018-3531-8

Asadishad B., Chahal S., Akbari A., Cianciarelli V., Azodi M., Ghoshal S., Tufenkji N. Amendment of Agricultural Soil with Metal Nanoparticles: Effects on Soil Enzyme Activity and Microbial Community Composition. (2018) *Environ Sci Technol.* Feb 20;52(4):1908-1918. doi: 10.1021/acs.est.7b05389. Epub 2018 Feb 8. Erratum in: Environ Sci Technol. 2021 Mar 16;55(6):4077-4078. PMID: 29356510.

Cao Y., Ma C., Chen H., Chen G., White J.C., Xing B., Copper stress in flooded soil: Impact on enzyme activities, microbial community composition and diversity in the rhizosphere of Salix integra. (2020) *Science of The Total Environment*, Volume 704, 135350, ISSN 0048-9697, https://doi.org/10.1016/j.scitotenv.2019.135350.

Carley L.N., Panchagavi R., Song X., Davenport S., Bergemann C.M., McCumber A.W., Gunsch C.K., Simonin M. Long-Term Effects of Copper Nanopesticides on Soil and Sediment Community Diversity in Two Outdoor Mesocosm Experiments. (2020) *Environ Sci Technol.* Jul 21;54(14):8878-8889. doi: 10.1021/acs.est.0c00510. Epub 2020 Jun 30. PMID: 32543178.

Cui J., Wang W., Peng Y., Zhou F., He D., Wang J., Chang Y., Yang J., Zhou J., Wang W., Yao D., Du F., Liu X., Zhao H. Effects of simulated Cd deposition on soil Cd availability, microbial response, and crop Cd uptake in the passivation-remediation process of Cd-contaminated purple soil. (2019) *Sci Total Environ.* Sep 15;683:782-792. doi: 10.1016/j.scitotenv.2019.05.292. Epub 2019 May 22. PMID: 31150898.

Ding Z., Wu J., You A., Huang B., Cao C. Effects of heavy metals on soil microbial community structure and diversity in the rice (*Oryza sativa* L. subsp. Japonica, Food Crops Institute of Jiangsu Academy of Agricultural Sciences) rhizosphere. (2017)  *Soil Science and Plant Nutrition*, 63:1, 75-83, DOI: [10.1080/00380768.2016.1247385](https://doi.org/10.1080/00380768.2016.1247385)

Ding Q., Huang X., Hu H., Hong M., Zhang D., Wang K. Impact of pyrene and cadmium co-contamination on prokaryotic community in coastal sediment microcosms. (2017) *Chemosphere*. Dec;188:320-328. doi: 10.1016/j.chemosphere.2017.08.124. Epub 2017 Aug 23. PMID: 28888120.

Duan C., Liu Y., Zhang H. Cadmium Pollution Impact on the Bacterial Community of Haplic Cambisols in Northeast China and Inference of Resistant Genera. (2020) *J Soil Sci Plant Nutr* **20,**1156–1170. https://doi.org/10.1007/s42729-020-00201-5

Guan X., Gao X., Avellan A., Spielman-Sun E., Xu J., Laughton S., Yun J., Zhang Y., Bland G.D., Zhang Y., Zhang R., Wang X., Casman E.A., Lowry G.V. CuO Nanoparticles Alter the Rhizospheric Bacterial Community and Local Nitrogen Cycling for Wheat Grown in a Calcareous Soil. (2020) *Environ Sci Technol.* Jul 21;54(14):8699-8709. doi: 10.1021/acs.est.0c00036. Epub 2020 Jul 9. PMID: 32579348.

Hou D., Wang R., Gao X., Wang K., Lin Z., Ge J., Liu T., Wei S., Chen W., Xie R., Yang X., Lu L., Tian S., Cultivar-specific response of bacterial community to cadmium contamination in the rhizosphere of rice (Oryza sativa L.). (2018) *Environmental Pollution*, Volume 241, Pages 63-73, ISSN 0269-7491, https://doi.org/10.1016/j.envpol.2018.04.121.

Hu L., Wang R., Liu X., Xu B., Xie T., Li Y., Wang M., Wang G., Chen Y. Cadmium phytoextraction potential of king grass (Pennisetum sinese Roxb.) and responses of rhizosphere bacterial communities to a cadmium pollution gradient. (2018) *Environ Sci Pollut Res* *Int.* Aug;25(22):21671-21681. doi: 10.1007/s11356-018-2311-9. Epub 2018 May 21. PMID: 29785604.

Keiblinger K.M., Schneider M., Gorfer M. Assessment of Cu applications in two contrasting soils—effects on soil microbial activity and the fungal community structure. (2018) *Ecotoxicology.* **27,**217–233. https://doi.org/10.1007/s10646-017-1888-y

Kou S., Vincent G., Gonzalez E., Pitre F.E., Labrecque M., Brereton N.J.B. The Response of a 16S Ribosomal RNA Gene Fragment Amplified Community to Lead, Zinc, and Copper Pollution in a Shanghai Field Trial. (2018) *Front Microbiol.* Mar 1;9:366. doi: 10.3389/fmicb.2018.00366. PMID: 29545788; PMCID: PMC5838024.

Li J., Ma YB., Hu HW., Wang JT., Liu YR., He JZ. Field-based evidence for consistent responses of bacterial communities to copper contamination in two contrasting agricultural soils. (2015) *Frontiers in Microbiology*. 6, , 31, 10.3389/fmicb.2015.00031, 1664-302X

Li J., Wang JT., Hu HW., Ma YB., Zhang LM., He JZ. Copper pollution decreases the resistance of soil microbial community to subsequent dry-rewetting disturbance. (2016) *J Environ Sci* (China). Jan;39:155-164. doi: 10.1016/j.jes.2015.10.009. Epub 2015 Dec 19. PMID: 26899654.

Li J., Liu YR., Cui LJ., Hu HW., Wang JT., He JZ. Copper Pollution Increases the Resistance of Soil Archaeal Community to Changes in Water Regime. (2017) *Microb Ecol.* Nov;74(4):877-887. doi: 10.1007/s00248-017-0992-0. Epub 2017 May 10. PMID: 28492987.

Lin H., Liu C., Li B., Dong Y. Trifolium repens L. regulated phytoremediation of heavy metal contaminated soil by promoting soil enzyme activities and beneficial rhizosphere associated microorganisms. (2021) *J Hazard Mater.* Jan 15;402:123829. doi: 10.1016/j.jhazmat.2020.123829. Epub 2020 Sep 2. PMID: 33254810.

Liu Y., Li Y., Pan B., Zhang X., Zhang H., Steinberg C.E.W., Hao Q., Vijver M.G., Peijnenburg W.J.G.M. Application of low dosage of copper oxide and zinc oxide nanoparticles boosts bacterial and fungal communities in soil. (2021) *Science of The Total Environment*, Volume 757, 143807, ISSN 0048-9697, https://doi.org/10.1016/j.scitotenv.2020.143807.

McTee M., Bullington L., Rillig M.C., Ramsey P.W. Do soil bacterial communities respond differently to abrupt or gradual additions of copper? (2019) *FEMS microbiology* ecology, 95(1), fiy212. https://doi.org/10.1093/femsec/fiy212

Moore J.D., Stegemeier J.P., Bibby K., Marinakos S.M., Lowry G.V., Gregory K.B. Impacts of Pristine and Transformed Ag and Cu Engineered Nanomaterials on Surficial Sediment Microbial Communities Appear Short-Lived. (2016) *Environ Sci Technol*. Mar 1;50(5):2641-51. doi: 10.1021/acs.est.5b05054. Epub 2016 Feb 18. PMID: 26841726.

Naveed M., Møldrup P.L., Arthur E., Holmstrup M., Nicolaisen M., Tuller M., Herath L., Hamamoto S., Kawamoto K., Komatsu T., Vogel H., Jonge L.W.. Simultaneous Loss of Soil Biodiversity and Functions along a Copper Contamination Gradient: When Soil Goes to Sleep. (2014) *Soil Science Society of America Journal, 78*, 1239-1250.

Niu H., Leng YF., Li X., Yu Q., Wu H., Gong J., Li HL., Chen K. Behaviours of cadmium in rhizosphere soils and its interaction with microbiome communities in phytoremediation. (2021) *Chemosphere*. Volume 269, 128765, ISSN 0045-6535, https://doi.org/10.1016/j.chemosphere.2020.128765.

Pu S.., Yan C, Huang H., Liu S., Deng D. Toxicity of nano-CuO particles to maize and microbial community largely depends on its bioavailable fractions. (2019) *Environ Pollut.* Dec;255(Pt 2):113248. doi: 10.1016/j.envpol.2019.113248. Epub 2019 Sep 17. PMID: 31561034.

Qiu Z., Yinghua Z., Xiu Z., Jing S. Effects of biochar on bacterial genetic diversity in soil contaminated with cadmium. (2021) *Soil Use Manage*. ; 37: 289– 298. 
https://doi.org/10.1111/sum.12678

Sarria Carabalí M.M., García-Oliva F., Cortés Páez L.E., López-Lozano N.E. Effect of cadmium contamination on the rhizosphere bacterial diversity of Echinocactus platyacanthus. *Rhizosphere*. (2020) Volume 13, 100187, ISSN 2452-2198, https://doi.org/10.1016/j.rhisph.2020.100187.

Sutcliffe B., Hose G.C., Harford A.J., Midgley D.J., Greenfield P., Paulsen I.T., Chariton A.A. Microbial communities are sensitive indicators for freshwater sediment copper contamination. (2019) *Environ Pollut*. Apr;247:1028-1038. doi: 10.1016/j.envpol.2019.01.104. Epub 2019 Jan 30. PMID: 30823331.

Wang S., Zhang L., Jiang M., Wang J., Xia F., Shi L., Xia Y., Chen C., Shen Z., Chen Y. Cyclic and safety utilization of Cu polluted biogas residue in saline-alkali soil. (2020) *Sci Total Environ.* Feb 20;704:135410. doi: 10.1016/j.scitotenv.2019.135410. Epub 2019 Nov 20. PMID: 31791757.

Wu B., Hou S., Peng D., Wang Y., Wang C., Xu F., Xu H.. Response of soil micro-ecology to different levels of cadmium in alkaline soil. (2018) *Ecotoxicology and Environmental Safety*, Volume 166, Pages 116-122, ISSN 0147-6513, https://doi.org/10.1016/j.ecoenv.2018.09.076.

Yang J., Xie Y., Jeppe K., Long S., Pettigrove V., Zhang X. Sensitive community responses of microbiota to copper in sediment toxicity test. (2018) *Environ Toxicol Chem.* Feb;37(2):599-608. doi: 10.1002/etc.3980. Epub 2017 Nov 30. PMID: 28892189.

Yin P., Liu X., Liao J., Hu X. Effects of Cadmium Stress on Microbial Community Diversity in Soil Potted With Sasa Argenteastriatus. (2019) IOP Conference Series: Earth and Environmental Science.

Zhang X., Xu Z., Qian X., Lin D., Zeng T., Filser J., Li L., Kah M. Assessing the Impacts of Cu(OH)_2_ Nanopesticide and Ionic Copper on the Soil Enzyme Activity and Bacterial Community. (2020) *J Agric Food Chem.* Mar 18;68(11):3372-3381. doi: 10.1021/acs.jafc.9b06325. Epub 2020 Mar 5. PMID: 32109358.

Zheng L., Li Y., Shang W. The inhibitory effect of cadmium and/or mercury on soil enzyme activity, basal respiration, and microbial community structure in coal mine–affected agricultural soil. (2019) *Ann Microbiol* **69,**849–859 https://doi.org/10.1007/s13213-019-01478-3
